# Discordance between pediatric self‐report and parent proxy‐report symptom scores and creation of a dyad symptom screening tool (co‐SSPedi)

**DOI:** 10.1002/cam4.3235

**Published:** 2020-06-21

**Authors:** Deborah Tomlinson, Erin Plenert, Grace Dadzie, Robyn Loves, Sadie Cook, Tal Schechter, Jennifer Furtado, L. Lee Dupuis, Lillian Sung

**Affiliations:** ^1^ Child Health Evaluative Sciences The Hospital for Sick Children Toronto ON Canada; ^2^ Department of Pharmacy The Hospital for Sick Children Toronto ON Canada; ^3^ Leslie Dan Faculty of Pharmacy University of Toronto Toronto ON Canada; ^4^ Division of Haematology and Oncology The Hospital for Sick Children Toronto ON Canada

**Keywords:** cancer, child, discordance, hematopoietic stem cell transplantation, SSPedi, symptoms

## Abstract

Symptom Screening in Pediatrics Tool (SSPedi) (age 8‐18 years) and mini‐SSPedi (age 4‐7 years) can be used to self‐report and proxy‐report bothersome symptoms in pediatric patients receiving cancer treatments. There are limitations of sole child self‐report or proxy‐report. An approach in which children and parents complete symptom reports together may be useful. The aim of our study was to describe discordance between child self‐report and parent proxy‐report symptom scores, and to determine how these scores compare to an approach in which reporting is performed together (co‐SSPedi). Children and parents completed SSPedi or mini‐SSPedi separately. Discordant symptoms were shared with respondents and discussed. Next, the dyad completed co‐SSPedi together and were asked which approach they preferred. Discordance was evaluated for each symptom and was defined as a difference of at least 2 points on an ordinal scale ranging from 0 (not at all bothered) to 4 (extremely bothered). Of the 48 enrolled dyads (children, median age, 10.8 years; 54.2% male), 41 (85.4%) had discordance in at least one symptom. There was no clear pattern in discordance by age group. When a dyad approach was used, more co‐SSPedi scores agreed with the original child self‐report scores (59 dyads, 56.2%) compared to original parent proxy‐report scores (15 dyads, 14.3%) for discordant symptoms. Forty‐three (89.6%) dyads preferred to complete SSPedi together. Future work should evaluate the psychometric properties of co‐SSPedi.

## Graphical abstract

Discordance is common between child self‐report and parent proxy‐report of symptom in children receiving cancer treatments, with no clear pattern in discordance by whether the symptom was subjective or objective, nor was there a clear pattern by age group. When a dyad approach was used, more scores agreed with the original child self‐report scores compared to original parent proxy‐report scores for discordant symptoms.

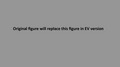

**Key points**

- In children receiving cancer treatments, there is often disagreement between symptom reports of child and parent.
- Using Symptom Screening in Pediatrics Tool (SSPedi) (8‐18 years) or mini‐SSPedi (4‐7 years), 48 child and parent dyads participated; 41 (85.4%) had discordance in at least one symptom (difference ≥2 points, 0‐4 scale).
- Using an approach where child and parent report together (co‐SSPedi), more scores agreed with original child self‐report scores (59 dyads, 56.2%) compared to original proxy‐report scores (15 dyads, 14.3%).
- Forty‐three (89.6%) dyads preferred to report symptoms together rather than separately.

## 1 BACKGROUND

Symptoms are common and often severely bothersome in pediatric patients receiving cancer treatments.^1^ In order to measure the extent of bothersome symptoms, the Symptom Screening in Pediatrics Tool (SSPedi) was developed. SSPedi has evidence for its reliability, validity, and responsiveness to change in pediatric patients aged 8‐18 years receiving cancer treatments.^2^, ^3^ Mini‐SSPedi was developed for children 4‐7 years and exhibits face and content validity.^4^ These instruments were developed to address the lack of appropriate symptom screening tools for these populations.^5^, ^6^, ^7^ They are available in both self‐report and proxy‐report formats.

Differences between child self‐report and parent proxy‐report quality of life scores have been well described in pediatric populations.^8^, ^9^ While some differences may arise due to measurement error, there has been increasing recognition that each reporter may have unique and valid perspectives.^10^ This has led to a suggestion to collect both child and parent report when possible.^10^ While these issues have been highlighted in the context of research, there is growing interest in incorporating patient‐reported outcomes into pediatric cancer clinical care and thus, the choice of reporter type needs to be considered in this setting as well.^11^, ^12^

When used in clinical care, obtaining both child and parent report will commonly not be feasible. There are settings in which children will not be willing to independently report symptoms, such as when they are very ill. Unfortunately, it is particularly in this setting that obtaining symptoms reports is crucial. While young children may be able to independently report symptoms on a single occasion in the context of a carefully conducted research study, they are less likely to be able to repeatedly and independently report their symptoms. Finally, the burden and logistical complexity of separate child and parent reporting would be associated with considerable challenges for clinical implementation. This burden is related to time and energies on behalf of the respondents, systems required to implement them, and the need for clinicians to view separate reports at each encounter.

In considering how routine symptom screening could be implemented into clinical practice, we hypothesized that a dyad approach, where SSPedi is completed by both the child and parent together, may be one way to address these challenges. Objectives of this study were to describe discordance between child self‐report and parent proxy‐report symptom scores, and to determine how these scores compare to an approach in which reporting is performed together (co‐SSPedi).

## 2 MATERIALS AND METHODS

This was a mixed methods study that included a quantitative and a qualitative component performed using a semi‐structured interview. This report consists of the quantitative responses and qualitative comments related to the completion of co‐SSPedi. The primary qualitative component focused on describing reasons for discordance will be reported later.

### 2.1 Subjects

Inclusion criteria were English‐speaking dyads of a child and a parent, where the child was between 4 and 18 years of age and had a diagnosis of cancer or was a hematopoietic stem cell transplant (HSCT) recipient. Exclusion criteria were illness severity, cognitive disability, or other impairment that precluded completion of SSPedi or mini‐SSPedi according to the primary healthcare team.

### 2.2 Procedures

Child and parent dyads were recruited from The Hospital for Sick Children (SickKids) in Toronto, Canada. The study received Research Ethics Board approval from SickKids, and participants provided informed consent or assent to participate. Potential dyads were approached in the inpatient and outpatient settings by a member of the study team. Sampling was consecutive within each age cohort and ensured even distribution by age group.

For all participants, demographic information was obtained from the respondent's parent and from the patient's health records.

Interviews were conducted by a clinical research nurse with experience in cognitive interviewing (DT), while a second team member recorded non‐verbal actions and additional field notes (EP, GD, or RL). First, the child and parent separately completed the self‐report and proxy‐report version of SSPedi or mini‐SSPedi on paper. Children 8‐18 years of age completed SSPedi, which is comprised of 15 symptoms rated on a 5‐point Likert degree of bother scale with a recall period of yesterday or today. Children 4‐7 years of age completed mini‐SSPedi, which is comprised of the same 15 symptoms rated on a 3‐point Likert degree of bother scale with a recall period of today. All parents completed the same proxy‐report SSPedi, which is identical to SSPedi (5‐point Likert scale with a recall period of yesterday or today), with the exception that questions were directed toward “your child” rather than “you.” The anchors (0= “not at all bothered” and 4= “extremely bothered”) and mid‐point (2= “medium amount of bother”) are identical between SSPedi, mini‐SSPedi, and proxy SSPedi. Respondents were instructed to complete the instrument independently, without discussion with the other reporter. If the child had questions about SSPedi or mini‐SSPedi, the clinical research nurse responded rather than the parent.

Once both the child and parent finished completing the self‐report and proxy‐report versions of SSPedi or mini‐SSPedi, the responses were shared with the dyad. We reassured them that inconsistencies are common with neither response being “wrong.” Viewing the two completed versions of SSPedi or mini‐SSPedi together, we compared each rater's score for each symptom one‐by‐one. If the difference in scores was 2 or more (on an ordinal scale that ranged from 0 to 4), this difference was voiced to the dyad. Starting with the child, we asked for their impression of potential reasons behind the difference. We then asked the same question to the parent. The same process was repeated for each symptom with a difference of 2 or more points between child and parent symptom scores. This portion of the interview was audiotaped and transcribed verbatim.

Next, we asked the dyad to complete co‐SSPedi together thinking about the knowledge they had gained from the preceding process. The dyads completed either SSPedi or mini‐SSPedi according to the child's age. The dyads were instructed to discuss each symptom in which their impression was different before arriving at a final symptom score. Any comments were noted and recorded by the second team member; this portion of the interview was not audiotaped. Finally, in each other's presence, the child and parent were asked whether they would prefer to complete SSPedi or mini‐SSPedi together or separately.

### 2.3 Outcome

The primary outcome was discordance. SSPedi and proxy‐SSPedi are scored as 0, 1, 2, 3, or 4 while mini‐SSPedi is scored as 0, 2, or 4. Consequently, SSPedi, proxy‐SSPedi, and mini‐SSPedi are all reported on an ordinal scale that ranges from 0 (not at all bothered) to 4 (extremely bothered). For both SSPedi and mini‐SSPedi, discordance between child and parent symptom report was defined as a difference of at least 2 points for any symptom.

While the primary qualitative analysis will focus on the reasons for discordance, we included qualitative comments regarding completion of co‐SSPedi in this report.

### 2.4 Analysis

We described the number of dyads with at least one discordant symptom and discordance stratified by age group (4‐7, 8‐10, 11‐14, and 15‐18) and symptom. As discordance will increase as the prevalence of a bothersome symptom increases, we described the percentage of dyads with discordance by symptom overall and among the subgroup of dyads in which either the child or parent reported any degree of bother. For dyads with discordance, we also reported which respondent reported worse bothersome symptoms.

Next, we reported whether co‐SSPedi resulted in the same scores as the original child self‐report version, the original parent proxy‐report version, or neither the original child nor parent versions. In the evaluation of the qualitative comments related to co‐SSPedi completion, two authors (DT and EP) independently coded the comments included in the field notes. They identified themes using thematic analysis.^13^, ^14^

We planned to enroll between 10 and 40 children in each age cohort of 4‐7, 8‐10, 11‐14, and 15‐18 years of age, ensuring we enrolled equal numbers to each cohort. Based on our previous research, we anticipated requiring up to 40 participants within each age group to reach saturation.^2^, ^4^, ^15^, ^16^, ^17^ We evaluated whether the interview process required modification and whether saturation had occurred for the primary qualitative aim after each group of 12 dyads. Review after each group of 12 allowed for even enrollment by age group (as it is divisible by 4). Saturation was defined as the absence of important new concepts or themes in the last group of 12 interviews.^18^

## 3 RESULTS

Between September 25, 2019 and January 19, 2020, 52 child‐parent dyads were assessed for eligibility. Two did not meet eligibility criteria and two declined, thus leaving 48 dyads who were enrolled and interviewed when saturation was achieved (12 in each age cohort). Table 1 describes the demographic characteristics of the child participants, highest parent education, and household income. Median age of the child was 10.8 years (range 4.8‐17.2). Among the parent participants, 34 (70.8%) were mothers.

**TABLE 1:** Demographic characteristics of participants (N = 48)

Characteristic	n (%)
Patient age in years	
4‐7	12 (25.0)
8‐10	12 (25.0)
11‐14	12 (25.0)
15‐18	12 (25.0)
Male sex	26 (54.2)
Diagnosis	
Leukemia or lymphoma	26 (54.2)
Solid tumor	16 (33.3)
Brain tumor	3 (6.3)
Other	3 (6.3)
Inpatient	10 (20.8)
On active treatment	41 (85.4)
Time since diagnosis in months	
≤6	20 (41.7)
>6‐12	14 (29.2)
>12	14 (29.2)
Relapse	14 (29.2)
Hematopoietic stem cell transplant	12 (25.0)
Second language^a^	26 (54.2)
Hindi	3 (6.3)
Mandarin	3 (6.3)
French	4 (8.3)
Other	21 (43.8)
Parent highest education	
High school	6 (12.5)
College or university	30 (62.5)
Professional or graduate	12 (25.0)
Household income	
<20 000	3 (6.3)
$20 000‐59 999	9 (18.8)
$60 000‐99 999	13 (27.1)
>$100 000	19 (39.6)
Undisclosed	4 (8.3)

^a^ Numbers do not add to total as some understood more than one second language.

Table 2 describes the symptoms reported as bothersome by either the child or parent. Symptoms most commonly reported as any degree of bother by either the child or parent were “feeling tired” (n = 42; 87.5%) and “feeling disappointed or sad” (n = 42; 87.5%). Symptoms least commonly reported as any degree of bother by either the child or parent were “mouth sores” (n = 9) and “diarrhea” (n = 12). Table 2 also describes the number of dyads with discordance by symptoms overall and stratified by age group. Forty‐one dyads (85.4%) had discordance in at least one symptom. Among all 720 symptoms reported (48 dyads reported on 15 symptoms), discordance occurred in 105/720 (14.6%). However, if restricted to the dyads in which either the child or parent reported any degree of bother, then discordance occurred in 105/433 (24.2%) of symptoms. Table 2 shows that there was no clear pattern by age group when considering the prevalence of discordance among those in which either the child or parent reported any degree of bother. The percentage of dyads with discordance among those who rated any bother ranged from 11.8% for “headache” to 50.0% for “diarrhea.”

**TABLE 2:** Discordance between self‐report and parent proxy‐report SSPedi or mini‐SSPedi scores and distribution by age

Symptom	Any bother by child or parent (N = 48)	Number with discordance^a^					
		Among all participants, n (%)	Among those with any bother, n (%)	Age 4‐7 y, (n = 12)	Age 8‐10 y, (n = 12)	Age 11‐14 y, (n = 12)	Age 15‐18 y, (n = 12)
Feeling scared or worried	36	11/48 (22.9%)	11/36 (30.6%)	4	5	1	1
Feeling more or less hungry than you usually do	35	11/48 (22.9%)		3	2	4	2
Changes in taste	30	11/48 (22.9%)	11/30 (36.7%)	2	2	2	5
Problems with thinking or remembering things	30	10/48 (20.8%)	10/30 (33.3%)	3	3	2	2
Feeling disappointed or sad	42	9/48 (18.8%)	9/42 (21.4%)	2	5	1	1
Feeling cranky or angry	36	9/48 (18.8%)	9/36 (25.0%)	4	2	1	2
Feeling tired	44	7/48 (14.6%)	7/44 (15.9%)	2	1	1	3
Changes in how you your body or face look	31	6/48 (12.5%)	6/31 (19.4%)	2	1	1	2
Hurt or pain (other than headache)	37	6/48 (12.5%)	6/37 (16.2%)	2	2	0	2
Throwing up or feeling like you may throw up	36	6/48 (12.5%)	6/36 (16.7%)	2	2	1	1
Diarrhea (watery, runny poop)	12	6/48 (12.5%)	6/12 (50.0%)	2	1	0	3
Constipation (hard to poop)	20	5/48 (10.4%)	5/20 (25.0%)	1	1	0	3
Mouth sores	9	3/48 (6.3%)	3/9 (33.3%)	0	2	1	0
Tingly or numb hands or feet	18	3/48 (6.3%)	3/18 (16.7%)	1	1	1	0
Headache	17	2/48 (4.2%)	2/17 (11.8%)	0	1	0	1
Total		105/720 (14.6%)	105/433 (24.2%)	30	31	16	28

Abbreviation: SSPedi, Symptom Screening in Pediatrics Tool.

^a^ Discordance defined as a difference of at least 2 points on an ordinal scale ranging from 0 = “not at all bothered” to 4 = “extremely bothered”.

Table 3 describes discordance and which reporter rated worse bothersome symptoms. There were more parents who reported worse bother for “feeling scared or worried” and “feeling disappointed or sad” compared with child self‐report. Conversely, there were more children who reported worse bother for “feeling more or less hungry than you usually do” and “changes in taste” compared with parent proxy‐report.

**TABLE 3:** Discordance between self‐report and parent proxy‐report SSPedi or mini‐SSPedi scores and which reporter rated worse bothersome symptoms

Symptom	Number with discordance all participants^a^	Number where child rated worse bother	Number where parent rated worse bother
Feeling scared or worried	11	1	10
Feeling more or less hungry than you usually do	11	7	4
Changes in taste	11	7	4
Problems with thinking or remembering things	10	6	4
Feeling disappointed or sad	9	0	9
Feeling cranky or angry	9	3	6
Feeling tired	7	4	3
Changes in how you your body or face look	6	3	3
Hurt or pain (other than headache)	6	3	3
Throwing up or feeling like you may throw up	6	3	3
Diarrhea (watery, runny poop)	6	4	2
Constipation (hard to poop)	5	3	2
Mouth sores	3	0	3
Tingly or numb hands or feet	3	1	2
Headache	2	0	2
Total	105	45	60

Abbreviation: SSPedi, Symptom Screening in Pediatrics Tool.

^a^ Discordance defined as a difference of at least 2 points on an ordinal scale ranging from 0 =”not at all bothered” to 4 = “extremely bothered”

Table 4 describes the results of co‐SSPedi completion and how these results compared to the original child self‐report and parent proxy‐report scores. Among the 105 symptoms with discordant scores, overall, more co‐SSPedi scores agreed with the original child self‐report scores (59 dyads, 56.2%) compared to the original parent proxy‐report scores (15 dyads, 14.3%). In 31 dyads (29.5%), co‐SSPedi was not the same as either the original child self‐report or parent proxy‐report scores and was intermediary between the original scores in 28/31 dyads.

**TABLE 4:** Relationship between Co‐SSPedi Score^b^ and initial child self‐report and parent proxy‐report symptom scores

Symptom	Number with discordance^a^	Co‐SSPedi score same as child score, n (%)	Co‐SSPedi score same as parent score, n (%)	Co‐SSPedi score neither child nor parent score, n (%)
Feeling scared or worried	11	9 (81.8%)	1 (9.1%)	1 (9.1%)
Feeling more or less hungry than you usually do	11	3 (27.3%)	3 (27.3%)	5 (45.4%)
Changes in taste	11	7 (63.6%)	0 (0.0%)	4 (36.3%)
Problems with thinking or remembering things	10	4 (40.0%)	1 (10.0%)	5 (50.0%)
Feeling disappointed or sad	9	5 (55.6%)	0 (0.0%)	4 (44.4%)
Feeling cranky or angry	9	4 (44.4%)	3 (33.3%)	2 (22.2%)
Feeling tired	7	4 (57.1%)	0 (0.0%)	3 (42.9%)
Changes in how you your body or face look	6	4 (66.7%)	1 (16.7%)	1 (16.7%)
Hurt or pain (other than headache)	6	3 (50.0%)	2 (33.3%)	1 (16.7%)
Throwing up or feeling like you may throw up	6	6 (100%)	0 (0.0%)	0 (0.0%)
Diarrhea (watery, runny poop)	6	3 (50.0%)	1 (16.7%)	2 (33.3%)
Constipation (hard to poop)	5	1 (20.0%)	2 (40.0%)	2 (40.0%)
Mouth sores	3	2 (66.7%)	0 (0.0%)	1 (33.3%)
Tingly or numb hands or feet	3	2 (66.7%)	1 (33.3%)	0 (0.0%)
Headache	2	1 (50.0%)	0 (0.0%)	1 (50.0%)
Total	105	59 (56.2%)	15 (14.3%)	31 (29.5%)

Abbreviation: SSPedi, Symptom Screening in Pediatrics Tool.

^a^ Discordance defined as a difference of at least 2 points on an ordinal scale ranging from 0 = “not at all bothered” to 4 = “extremely bothered”.

^b^ Co‐SSPedi score determined by child and parent together.

While observing the dyad completion of co‐SSPedi, in 41 dyads, the parent read each question aloud, in 4 dyads, the child read each question aloud, and in 3 dyads both parent and child each read some of the questions aloud. In 47 dyads, dialogue occurred between the two respondents for at least one symptom. Discussion often focused on the meaning of bother (13 dyads) and whether a symptom was bothersome within the recall period timeframe (8 dyads).

Forty‐three (89.6%) dyads preferred to complete SSPedi together. Of the five dyads who preferred completion separately, three children preferred to do SSPedi alone. In one of these three children, the parent expressed a preference for completing SSPedi together. In the remaining two dyads, parents preferred SSPedi completion separately because they were interested in seeing the different perspectives of child and parent.

There were four themes identified related to the completion of co‐SSPedi generated during its completion or when asked about preferences to complete SSPedi together or apart. The first theme focused on communication. Completion of co‐SSPedi facilitated discussion between child and parent. One parent reported, “I learned lots of new things, I have to communicate regularly about changes happening. Things are so fluid that it's a good thing to always ask.” The second theme related to strong relationships. Those with strong relationships were more likely to have previously discussed symptoms. The third theme referred to willingness or lack of willingness to share experienced symptoms. While many children were open about sharing, some were reluctant to share this information. Disinclination to share could be related to reluctance to communicate with their parent. Others also voiced that if they did not like the treatment for the symptom (for example laxatives for constipation), they were more hesitant to disclose their symptom experience. The final theme related to the distinction between presence vs degree of bother associated with a symptom. They noted that co‐SSPedi completion illuminated this distinction, particularly if a symptom was present but it was not bothersome.

## 4 DISCUSSION

We found that discordant symptom reporting was common, with no clear pattern in discordance by age group. When a dyad approach was used, more co‐SSPedi scores agreed with the original child self‐report scores compared to original parent proxy‐report scores. In addition, co‐SSPedi often led to symptom scores that were neither the child's nor the parent's original scores. Almost all participants preferred to complete SSPedi together.

We found that where discordance existed, parents tended to rate worse bother for “feeling disappointed and sad,” “feeling scared or worried,” and “feeling cranky or angry” compared to their child, while children tended to rate worse bother for “feeling more or less hungry than you usually do” and “changes in taste.” These are all subjective symptoms and thus, this finding is not consistent with others who have suggested that parents tend to report worse quality of life in general for subjective symptoms.^19^, ^20^ Previous studies have reported an association between parent quality of life and how they report their child's quality of life.^21^, ^22^ Thus, more sadness, worry, and anger reported by parents may reflect these same symptoms in the parent themselves. Alternatively, sadness, worry, and anger are more perceptible phenomenon whereas changes in hunger and taste may either be less perceptible, or may be less valued as important symptoms from the parent perspective. Another possibility is that children may fail to recall sadness, worry, and anger even within the short recall timeframe of SSPedi or mini‐SSPedi as these symptoms may be transient. Conversely, changes in hunger and taste may be more constant in nature, facilitating recall from the child perspective.

We took a dyad approach to symptom reporting in this study. There may be a concern that children would repress these symptoms, or conceal them to avoid burdening their parents or because they would not want their parents to know about them during co‐SSPedi completion.^23^ However, our results were not congruent with this concern since more co‐SSPedi scores were consistent with the original child SSPedi or mini‐SSPedi scores compared to the original parent scores. It is interesting that the discussion in completing co‐SSPedi often focused on the concept of bother or the clarification of the recall period. This may suggest that co‐SSPedi could result in more valid answers compared to self‐report alone for younger children who may have more challenges in understanding aspects of the symptom assessment tool.

While the creation of a dyad symptom report instrument for pediatric cancer patients is novel, the concept is not new. In the research setting, a dyad approach was successfully used to assess quality of life in children with asthma.^24^ In the pediatric cancer setting, a review of the perspectives of symptom reporting by children and family caregivers highlighted the complexity of symptom reporting.^25^ This review recognized that among pediatric cancer patients, parents often may need to either complete or assist in the completion of symptom reports.^25^, ^26^ There may also be other advantages of a dyad approach to symptom reporting. Baggott and colleagues (2015) suggested that sharing symptom reports could help in the communication of symptoms and provide a better understanding of perspectives between child and parent.^27^

It is important to emphasize that the recall periods of SSPedi and proxy‐SSPedi (regardless of age) differ compared with mini‐SSPedi. Because we found that younger children did not understand the concept of “yesterday,” mini‐SSPedi has a recall period of “today” while SSPedi and proxy‐SSPedi have a recall period of “yesterday or today.”^4^ This difference biases toward more discordance in younger children if bothersome symptoms occurred “yesterday” but not “today.” Because we wanted to ensure younger children could more fully participate in completion of co‐SSPedi, the version of co‐SSPedi for children 4‐7 years of age has a recall period of “today.” While this may limit comparability between SSPedi and mini‐SSPedi, we felt that optimizing the child's understanding of the symptom assessment tool was a more important factor to consider.

The strength of this study is the novel approach to symptom assessment for pediatric patients receiving cancer treatments that allows capture of the child's perspective while preserving feasibility. Co‐SSPedi may be an approach more amenable to clinical implementation rather than relying solely upon child self‐report or parent proxy‐report. A second strength is the description of how co‐SSPedi scores compared to either self‐report or proxy‐report symptom scores.

However, this study has several potential limitations. First, it was conducted at only one site that is a tertiary care pediatric cancer center in a high‐income country. It is important for future research to be conducted at additional sites to improve generalizability of the results. Second, the comparison of scores between mini‐SSPedi and proxy SSPedi (ages 4‐7 years) may have been affected by the different number of response options (3 for mini‐SSPedi and 5 for proxy SSPedi) although patterns did not clearly differ relative to the comparison between SSPedi and proxy SSPedi (ages 8‐18 years). Third, in this study, co‐SSPedi was completed after each respondent completed SSPedi independently following by discussion of discrepancies. Completion of co‐SSPedi by dyads as performed in this study may not be the same experience as naïve dyads who complete co‐SSPedi for the first time. Thus, while we found more co‐SSPedi scores were consistent with original child SSPedi or mini‐SSPedi scores compared to original parent scores, this finding may not have been true if respondents had not seen each other's scores nor discussed discrepancies. Fourth is the potential for bias when asked about preference to complete SSPedi together or separately, as both child and parent were present together when asked this question. A final limitation is that the sample size was too small to permit statistical comparison of mini‐SSPedi vs SSPedi results.

In conclusion, discordant reporting of bothersome symptoms was common. When symptom reporting was performed together (co‐SSPedi), the dyad approach more often agreed with the original child rather than parent scores. Future work should evaluate the psychometric properties of co‐SSPedi.

## CONFLICTS OF INTEREST

The authors declare no conflict of interest.

## AUTHOR CONTRIBUTIONS

All authors participated in the different aspects of the study. Overall concept and design of the study: DT, EP, LD, and LS. Operational aspects of the study: DT, EP, GD, RL, SC, and JF. Analysis and interpretation of the results: DT, EP, RL, SC, TL, LD, and LS. Study report first and last draft: DT and LS. All authors reviewed and amended the study manuscript repeatedly and approved the final version.

## Data Availability

Full details of the data produced and analyzed during this study are available from the corresponding author on reasonable request.
